# Vehicle and Pedestrian Traffic Signal Performance Measures Using LiDAR-Derived Trajectory Data

**DOI:** 10.3390/s24196410

**Published:** 2024-10-03

**Authors:** Enrique D. Saldivar-Carranza, Jairaj Desai, Andrew Thompson, Mark Taylor, James Sturdevant, Darcy M. Bullock

**Affiliations:** 1 Joint Transportation Research Program, Lyles School of Civil and Construction Engineering, Purdue University, West Lafayette, IN 47907, USA; desaij@purdue.edu (J.D.); thomp907@purdue.edu (A.T.); darcy@purdue.edu (D.M.B.); 2 Utah Department of Transportation, Traffic Operations Center, 2060 S 2760 W, Salt Lake City, UT 84104, USA; marktaylor@utah.gov; 3 Indiana Department of Transportation, Traffic Management Center, 8620 East 21st St., Indianapolis, IN 46219, USA; jsturdevant@indot.in.gov

**Keywords:** LiDAR, traffic signal, performance, vehicle, pedestrian

## Abstract

Light Detection and Ranging (LiDAR) sensors at signalized intersections can accurately track the movement of virtually all objects passing through at high sampling rates. This study presents methodologies to estimate vehicle and pedestrian traffic signal performance measures using LiDAR trajectory data. Over 15,000,000 vehicle and 170,000 pedestrian waypoints detected during a 24 h period at an intersection in Utah are analyzed to describe the proposed techniques. Sampled trajectories are linear referenced to generate Purdue Probe Diagrams (PPDs). Vehicle-based PPDs are used to estimate movement level turning counts, 85th percentile queue lengths (85QL), arrivals on green (AOG), highway capacity manual (HCM) level of service (LOS), split failures (SF), and downstream blockage (DSB) by time of day (TOD). Pedestrian-based PPDs are used to estimate wait times and the proportion of people that traverse multiple crosswalks. Although vehicle signal performance can be estimated from several days of aggregated connected vehicle (CV) data, LiDAR data provides the ability to measure performance in real time. Furthermore, LiDAR can measure pedestrian speeds. At the studied location, the 15th percentile pedestrian walking speed was estimated to be 3.9 ft/s. The ability to directly measure these pedestrian speeds allows agencies to consider alternative crossing times than those suggested by the Manual on Uniform Traffic Control Devices (MUTCD).

## 1. Introduction

Traffic signals have a significant impact on road transportation networks. It is estimated that up to 10% of all traffic delays on the National Highway System are a result of traffic signal operations. This represents a cost of tens of billions of dollars [1]. The National Transportation Operations Coalition (NTOC) indicates that a properly designed and managed traffic signal can reduce congestion, enhance mobility, decrease delays, and reduce the number of vehicle stops, therefore reducing fuel consumption and air pollutants [2]. With over 400,000 traffic signals in operation in the United States, it is important for transportation agencies to systematically monitor signal performance [3] and identify locations where signal retiming [4,5,6,7,8] or capital investments [9,10] could improve operations.

Currently, there are two popular datasets used to monitor traffic signal performance: high-resolution controller event data [11] and connected vehicle (CV) trajectory data [7]. Traffic signal high-resolution (i.e., tenth-of-a-second) controller event data (i.e., changes in detector outputs and signal states) are used to create visualizations and tools that evaluate signal performance known as Automated Traffic Signal Performance Measures (ATSPMs) [11,12]. Due to the data’s high reporting frequency, ATSPMs provide actionable insights that practitioners can use in real-time. However, ATSPMs are highly dependent upon detection configuration [13] and are sensitive to traffic conditions [14,15].

Alternatively, commercial CV trajectory data can also be used to derive scalable traffic signal performance measures [7,16,17,18]. These techniques provide accurate average performance estimations [14,15] since the entire experience of traversing vehicles can be analyzed. However, this dataset can only be utilized to evaluate trends due to relatively low market penetration rates (MPRs) [19] that require data aggregation over several days [7]. Furthermore, only vehicle data are usually available, making the analysis of other modes of transportation, such as pedestrians, impossible without additional datasets.

Cost-effectiveness benefits, as well as an increase in knowledge and acceptance, have sparked a growth of Light Detection and Ranging (LiDAR) technology usage across state transportation agencies [20,21] for different purposes [22,23,24,25,26,27,28]. LiDAR sensors estimate the relative location of objects and surfaces by calculating the delay of laser signals [29]. By fusing the detection of various LiDAR sensors [30,31], almost all objects traversing the equipped intersection can be accurately tracked over large distances with reporting frequencies that match those of ATSPMs. Additionally, LiDAR is capable of differentiating pedestrians and vehicles [32,33].

The LiDAR capabilities make it a viable option to obtain path-based trajectories for nearly 100% of vehicles and pedestrians at signalized intersections. So far, most studies have focused on the use of LiDAR to analyze roadway safety [28,34,35,36] or emulate loop detector calls. However, with the generation of complete high-resolution object trajectories that traverse the intersection, accurate real-time trajectory-based traffic signal performance measures that can capture trends and rare events under almost any traffic condition can also be calculated [37].

### 1.1. Motivation and Objective

Previous studies have estimated delay [38] and operational performance measures [37] at signalized intersections. Those studies focused on the performance evaluation of a single vehicle intersection movement and did not evaluate the performance variation by time of day (TOD). However, a performance analysis by TOD for all relevant movements at a signalized intersection is needed to provide practitioners with actionable knowledge of the operational conditions at their managed locations [7,10].

The objective of this study is to provide techniques to generate Purdue Probe Diagrams (PPDs) from LiDAR-derived vehicle and pedestrian trajectories to evaluate intersection performance by 15 min periods throughout a day. Vehicle PPDs are time-space diagrams that are used to estimate turning counts, 85th percentile queue lengths (85QL), arrivals on green (AOG), highway capacity manual (HCM) level of service (LOS) [39], split failures (SF), and downstream blockage (DSB) [7] for each relevant intersection movement. Pedestrian-based PPDs are time-space diagrams that are used to estimate wait times, and the proportion of people who traverse one or two crosswalks.

Additionally, LiDAR data also provides valuable information on pedestrian speeds when crossing the intersection’s crosswalks. This paper also analyzes the distribution of such speeds and compares them with current guidance provided by the Manual of Uniform Traffic Control Devices (MUTCD) [40]. The results can help practitioners verify the instructions from the MUTCD.

### 1.2. Paper Structure

To achieve the outlined objectives, the paper is organized as follows:

- The Methods section first provides context on the LiDAR data used in the study. Then, the techniques to create vehicle and pedestrian PPDs, from which performance estimations can then be derived, are discussed.
- The Results section first presents the movement level vehicle traffic signal performance measures by movement and by TOD. Then, the estimated pedestrian traffic signal performance is provided. Finally, the distribution of pedestrian speeds within the intersection’s crosswalks is evaluated.
- The Discussion and Conclusions section highlights the most relevant points discussed in the manuscripts.

## 2. Methods

This section describes the data, concepts, and techniques used to generate vehicle and pedestrian PPDs. The production of PPDs is critical as they allow for the estimation of trajectory-based traffic signal performance measures [7].

### 2.1. LiDAR Trajectory Data

This study uses two-dimensional LiDAR data of tracked objects classified as vehicles or pedestrians collected at a signalized intersection managed by the Utah Department of Transportation (UDOT) during a 24 h period starting on 16 April 2024, at 06:22:26 h. The dataset was derived from a third-party perception software that uses three-dimensional point cloud data [29] generated from four LiDAR sensors installed at the studied location.

The dataset consists of waypoints that represent the centroids of detected objects with a tenth-of-a-second sampling frequency. Each waypoint includes the following information: timestamp, x-position, y-position, speed, heading, object type (i.e., pedestrian or vehicle), and a unique object identifier. By chronologically linking individual waypoints with the same identifier, the estimated trajectory of a traversing object can be obtained. Therefore, a LiDAR-derived trajectory T of a traversing object is defined as the set of its waypoints $W_{i}$, with *i* = 1, 2, …, *k*, where *i* = 1 is the first and *i = k* is the last collected sample of the object. That is:

(1) $$T={\left\{W_{i}\right\}}_{i=1}^{k}$$

(2) $$W_{i}=\{identifier,\;type,\;{timestamp}_{i},{\mathrm{x-position}}_{i},{\mathrm{y-position}}_{i},\;{speed}_{i},\;{heading}_{i}\}$$

The *type* waypoint attribute is used to determine whether the object is a vehicle or a pedestrian.

**Figure 1:**
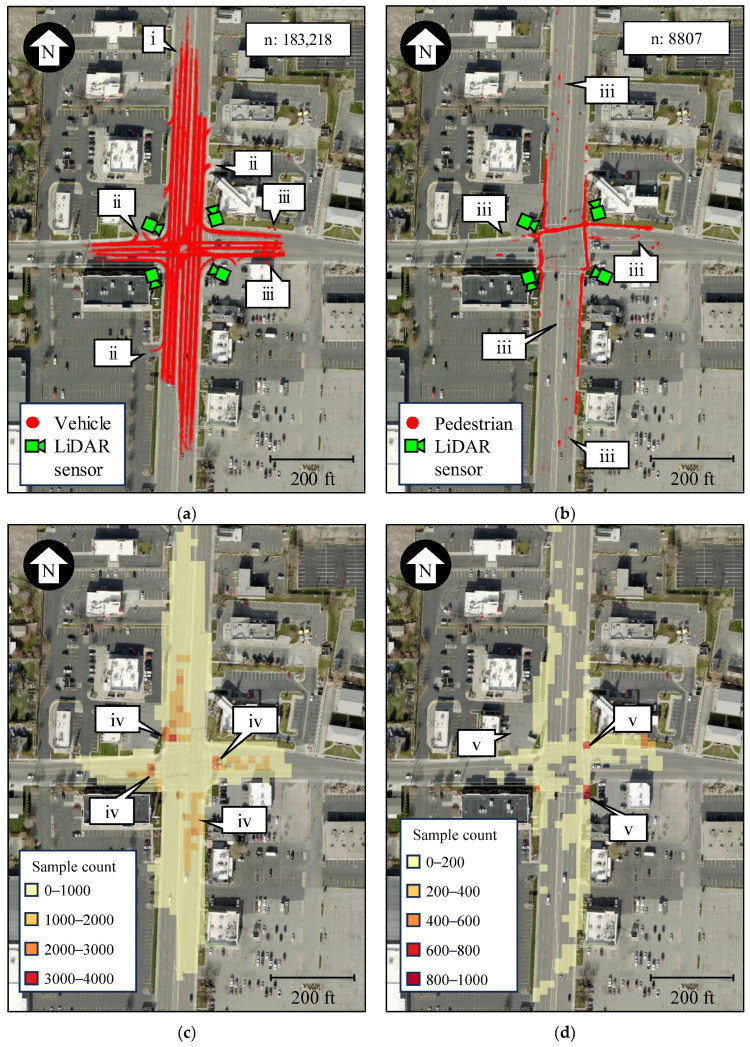
Tracked objects with LiDAR at State St. and 5900 S (map data: Esri, i-cubed, USDA, USGS, AEX, GeoEye, Getmapping, Aerogrid, IGN, IGP, UPR-EGP, and the GIS User Community). (**a**) Vehicles detected during a 5-min period. (**b**) Pedestrians detected during a 1-h period. (**c**) Vehicle intersection usage during a 5-min period. (**d**) Pedestrian intersection usage during a 1-h period.

Figure 1a,b show LiDAR-derived trajectories of vehicles and pedestrians at State St. and 5900 S in Salt Lake City. The quality of the data is such that sampled trajectories can be differentiated at the lane level (callout i). Objects are only detected when located on the roadways or sidewalks, and their movement within parking lots is ignored (callout ii).

It is important to note that, as with other detection systems, noise, and object misclassification can occur (callout iii). For this reason, techniques that only keep relevant trajectories for analysis are provided in subsequent sections. Effective approaches to verify the validity of sampled waypoints with LiDAR are presented in [37]. Additionally, this paper assumes that object classification is accurate and that further investigation is out of the scope of this study. This is because the objective of this manuscript is to provide the reader with techniques to derive actionable traffic signal performance measures, and the validity of derived trajectories at the analyzed intersection is irrelevant.

By converting the geospatial vector data shown in Figure 1a,b into rasters that summarize the number of detected samples, vehicle (Figure 1c) and pedestrian (Figure 1d) intersection usage can be evaluated. As expected, more vehicle samples are detected near the intersection’s stop bars (callout iv), where larger orange and red areas likely indicate larger queues. Similarly, more pedestrian waypoints are detected near the crosswalk corners (callout v), where a darker red indicates that people likely experienced larger wait times at those locations.

It is difficult to extract actionable insights from the raw vector and raster data (Figure 1). The following subsections describe how the LiDAR-derived object trajectories can be turned into vehicle and pedestrian PPDs, which will then be used in the Results section to estimate traffic signal operational performance measures.

### 2.2. Vehicle Analysis

Movement-level vehicle traffic signal performance measures can be calculated from LiDAR-derived vehicle trajectories by following these steps:

1. Identify the movement followed by sampled vehicles and filter irrelevant waypoints.
2. Construct PPDs from which relevant events and conditions can be estimated.
3. Evaluate 15 min of trajectory data in the same PPD by movement to estimate the intersection’s operational performance measures for that specific TOD period.

These steps are further described below.

#### 2.2.1. Movement Identification

It is crucial to estimate movement level traffic signal performance measures so that the interactions, challenges, and opportunities between signal phases can be evaluated [7]. Therefore, sampled vehicle trajectories need to be assigned one of the following relevant intersection movements: southbound-left (SBL), northbound-through (NBT), westbound-left (WBL), eastbound-through (EBT), northbound-left (NBL), southbound-through (SBT), eastbound-left (EBL), and westbound-through (WBT). In certain cases, right-turn movements need to be analyzed, but in most intersections, they are not a significant factor.

In this study, a sampled vehicle trajectory is identified as having followed a particular intersection movement when it first crosses the movement’s stop bar and then the movement’s far side (FS). If additional stop bars or far sides are crossed, or if they are crossed in the incorrect order, that vehicle trajectory is not assigned an intersection movement and is therefore excluded from further analysis.

**Figure 2:**
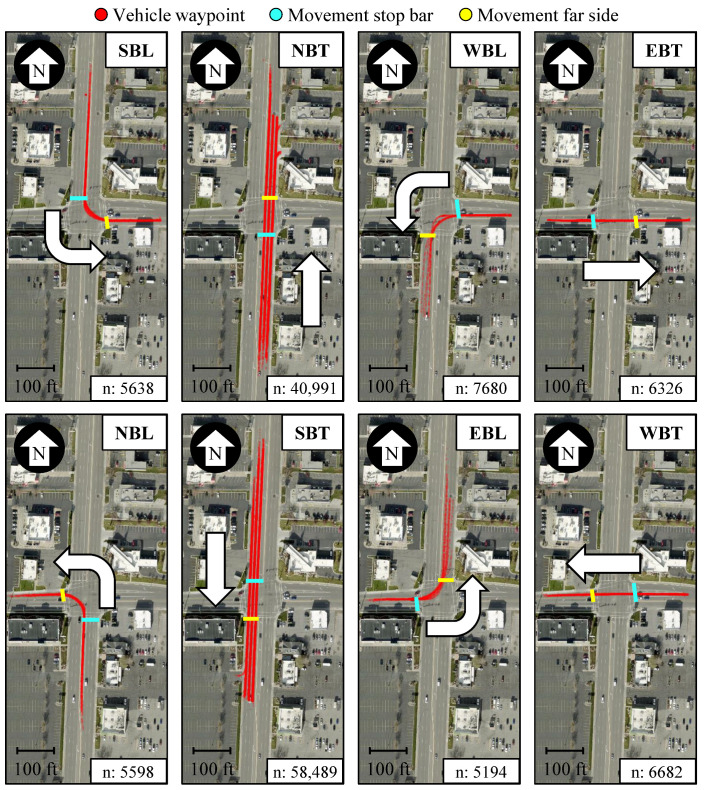
Detected vehicles following relevant intersection movements during a 5 min period (map data: Esri, i-cubed, USDA, USGS, AEX, GeoEye, Getmapping, Aerogrid, IGN, IGP, UPR-EGP, and the GIS User Community).

Figure 2 shows LiDAR-derived vehicle trajectories sampled during a 5 min period identified as having followed one of the eight relevant movements. In each of these cases, the trajectories shown first crossed the assigned movement’s stop bar (blue) and then the same movement’s far side (yellow). This approach assigns intersection movements to traversing vehicles and makes sure that the trajectories kept for further analysis traveled at least the length of the intersection. This also filters noise detection that is usually sampled at one stop or over short distances.

#### 2.2.2. Vehicle PPD Concepts

PPDs can be constructed once vehicle trajectories are assigned an intersection movement. A PPD is a time-space diagram with color-coded trajectory segments that show the experience of traversing vehicles. The vertical axis indicates the distance, and the horizontal axis indicates the time, for a vehicle to reach the far side (FS) of its movement. A free-flow trajectory (FFT) of a hypothetical vehicle traveling unimpeded at the posted speed limit is plotted for reference. In a vehicle PPD, the estimation of the following performance measures is facilitated [7]:

- 85QL: the 85th percentile queue length of a particular movement provides information on whether storage and detection areas are large enough. The length of a queue is estimated as the distance to the far side when an approaching vehicle first stops. Queues that reach the detection limits indicate that other performance estimations may be inaccurate [7,13,15].
- LOS: this classification is based on control delay thresholds as provided in Table 1 [39]. In a PPD, the control delay $d_{cT}$ experienced by a sampled trajectory T is calculated as:

(3) $$d_{cT}=TT_{T}-TT_{FFT}$$

where $TT_{T}$ is the travel time (s) of T and $TT_{FFT}$ is the travel time (s) of the FFT. The average control delay (s/veh) of an intersection movement during a given TOD period with *n* sampled trajectories is calculated as:

(4) $$\mathrm{Average}\;\mathrm{Control}\;\mathrm{Delay}=\frac{1}{n}\sum_{i=1}^{n}d_{CT_{i}}$$

with *i* = 1, 2, …, *n*, where *i* = 1 is the first and *i = n* is the last sampled trajectory during that TOD period.

- AOG: this operational measurement indicates the quality of progression by providing the percentage of vehicles that do not stop as they approach the intersection.
- SF: this operational measurement communicates the level of congestion at an intersection. It provides the percentage of vehicles that stopped at least twice before crossing the intersection that also had a control delay larger than any other vehicle that stopped less than twice for the same movement and TOD period.
- DSB: this operational measurement indicates the level of obstruction by an adjacent intersection. DSB is calculated as the percentage of vehicles that experience at least 10 s of delay after crossing the analyzed intersection.

**Figure 3:**
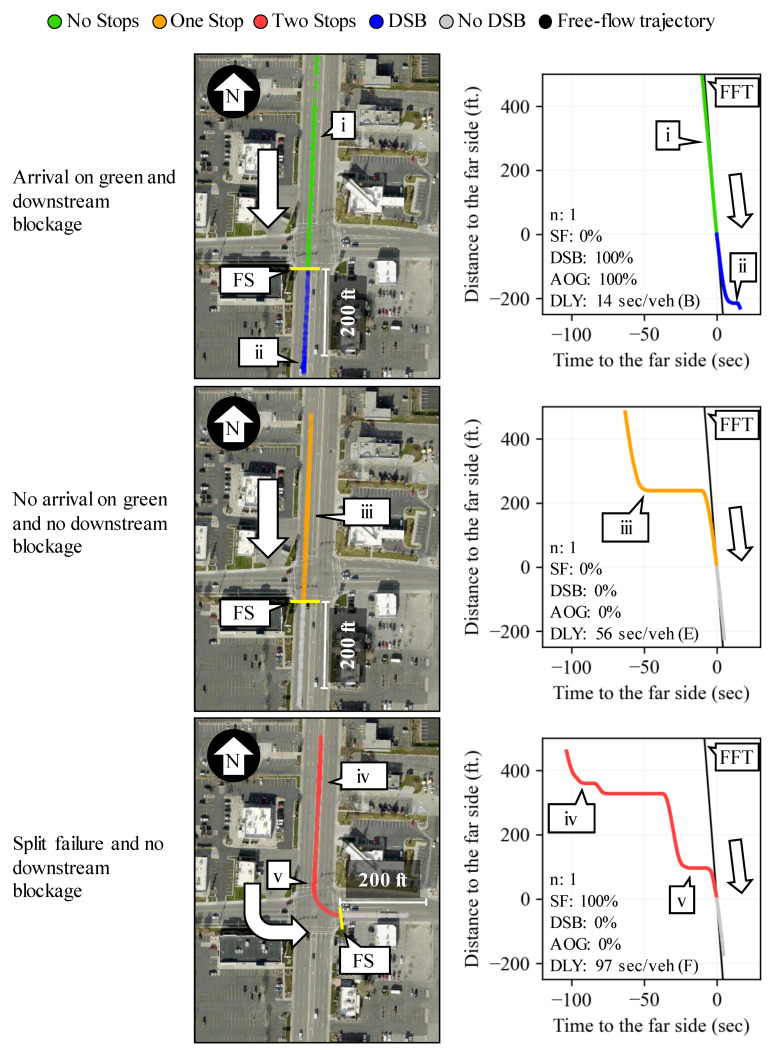
Geospatial and PPD representation of relevant vehicle performance events (map data: Esri, i-cubed, USDA, USGS, AEX, GeoEye, Getmapping, Aerogrid, IGN, IGP, UPR-EGP, and the GIS User Community). (Note: DLY = delay).

Figure 3 shows examples of PPDs (right) and the geospatial representations (left) of three vehicle trajectories with different experiences as they traveled through the intersection. The first analyzed trajectory (top) did not stop during its approach to the intersection (callout i); therefore, it is categorized as an arrival on green. However, it experienced a downstream blockage event just 200 ft. after crossing the far side (callout ii).

The second analyzed trajectory (middle) stopped once 225 ft. upstream of the far side (callout iii). For this reason, this vehicle is categorized as not an arrival on green. Since this vehicle only stopped once, the queue length for this movement is estimated to be 225 ft. from the far side at the time the vehicle came to a stop.

The third analyzed trajectory (bottom) stopped first 370 ft. (callout iv) and then 95 ft. (callout v) upstream of the far side. This vehicle is categorized as having experienced a split failure event because it stopped twice and had a control delay larger than any other evaluated trajectory for the same movement and TOD period. Additionally, the queue length for this movement is estimated to be 370 ft. from the far side at the time this vehicle first came to a stop.

#### 2.2.3. Vehicle PPD Production by TOD Period

**Figure 4:**
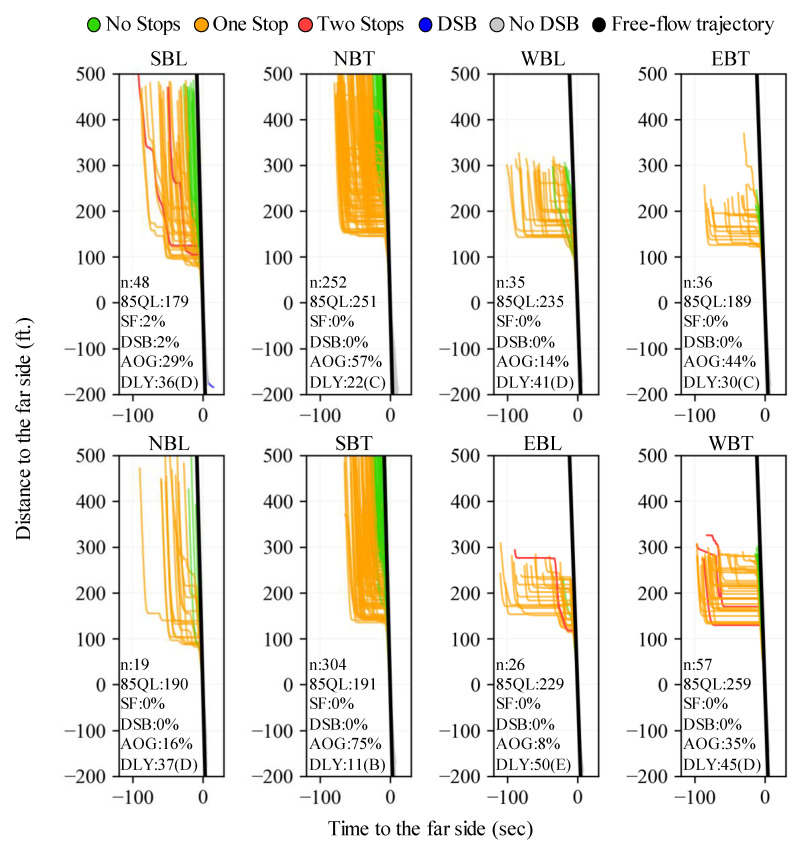
Vehicle PPDs of relevant intersection movements from 15:15 to 15:30 h. (Note: DLY = delay).

Once crossing trajectories are assigned an intersection movement, and their individual experience is quantified, entire intersection movements can be evaluated over a particular TOD period using all the sampled vehicle trajectories during that time. Figure 4 shows PPDs and estimated signal performance for data collected during a 15 min period for all relevant movements. It is shown how all movements operate at a LOS below F, and how the NBT and SBT movements have the highest AOG values even though they handle the highest demands. The 24 h performance results by 15 min periods and movement are presented in the Results section.

### 2.3. Pedestrian Analysis

Intersection-level pedestrian traffic signal performance measures can be calculated from LiDAR-derived pedestrian trajectories by following these steps:

1. Construct PPDs from which relevant events and conditions can be estimated.
2. Evaluate all trajectory data during a TOD period to estimate the intersection’s performance for that specific TOD period.

These steps are further described below.

#### 2.3.1. Pedestrian PPD Concepts

A pedestrian PPD is a time-space diagram that shows the experience of traversing pedestrians color-coded based on the number of times a person crosses a crosswalk. The vertical axis indicates the distance, and the horizontal axis indicates the time, for a pedestrian to reach the center of the last crosswalk crossed. This facilitates the evaluation of pedestrians’ trips before and after they cross the intersection. Additionally, an FFT of a hypothetical person traveling unimpeded at 3.5 ft/s, as suggested in the MUTCD [40], is plotted for reference. Two performance measures that are estimated with this approach are:

- Wait time: this measurement indicates the total amount of time $WT_{P}$ (s) a pedestrian P waits before traveling through the crosswalk. The average pedestrian wait time (s/ped) at an intersection during a given TOD period with *n* sampled trajectories is calculated as:

(5) $$\mathrm{Average}\;\mathrm{Wait}\;\mathrm{Time}=\frac{1}{n}\sum_{i=1}^{n}WT_{P_{i}}$$

with *i* = 1, 2, …, *n*, where *i* = 1 is the first and *i = n* is the last sampled trajectory during that TOD period.

- Number of crosswalks crossed: this measurement indicates the percentage of pedestrians that crossed either one or two crosswalks. This information is relevant as it may help explain wait time estimations and can also be used to design signal timing [41,42].

**Figure 5:**
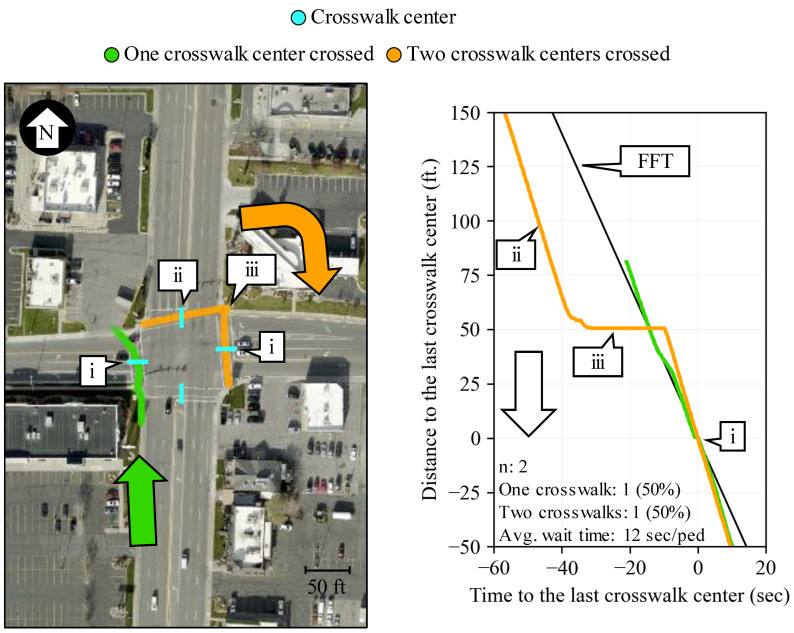
Geospatial and PPD representation of relevant pedestrian performance events (map data: Esri, i-cubed, USDA, USGS, AEX, GeoEye, Getmapping, Aerogrid, IGN, IGP, UPR-EGP, and the GIS User Community).

Figure 5 shows the geospatial (left) and PPD (right) representation of two trajectories of pedestrians that traversed the intersection. The green trajectory only crossed through one crosswalk (callout i), while the orange trajectory traversed two (callout ii and i). Their PPD representation pivots on the time and space where they passed the center of the last crosswalk crossed (callout i). The green trajectory did not experience any wait time. The orange trajectory had to wait 24 s (callout iii) after crossing its first crosswalk (callout ii) and before crossing its last crosswalk (callout i). Using Equation (4), the average intersection pedestrian wait time for the PPD analyzed in Figure 5 is 12 s/ped.

#### 2.3.2. Pedestrian PPD Production by TOD Period

**Figure 6:**
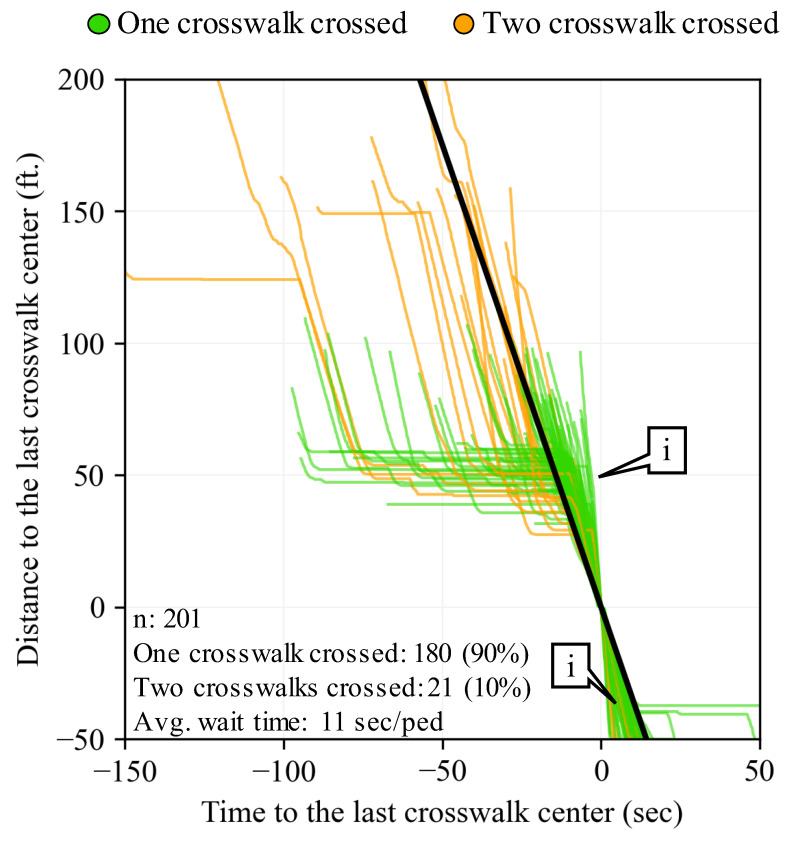
Pedestrian PPD over a 24 h period.

Once the individual experience of traversing pedestrians can be quantified, the entire intersection performance can be evaluated over a TOD period using all the sampled trajectories during that time. Figure 6 shows the PPD and estimated signal performance for data collected during the entire 24 h period. It is shown how most people, when actively walking, travel faster than the FFT that moves at 3.5 ft/s (callout i). Almost all pedestrians, 90%, only crossed one crosswalk. Additionally, on average, there is only an 11 s/ped wait time for the entire intersection.

The Results section presents performance estimations by 15 min TOD periods derived from the concepts discussed in this section.

## 3. Results

This section presents traffic signal performance measures results by 15 min TOD periods, first for vehicles and then for pedestrians. Lastly, pedestrian speeds within the crosswalks are evaluated and compared with current design guidelines.

### 3.1. Vehicle Performance by Movement and TOD

**Figure 7:**
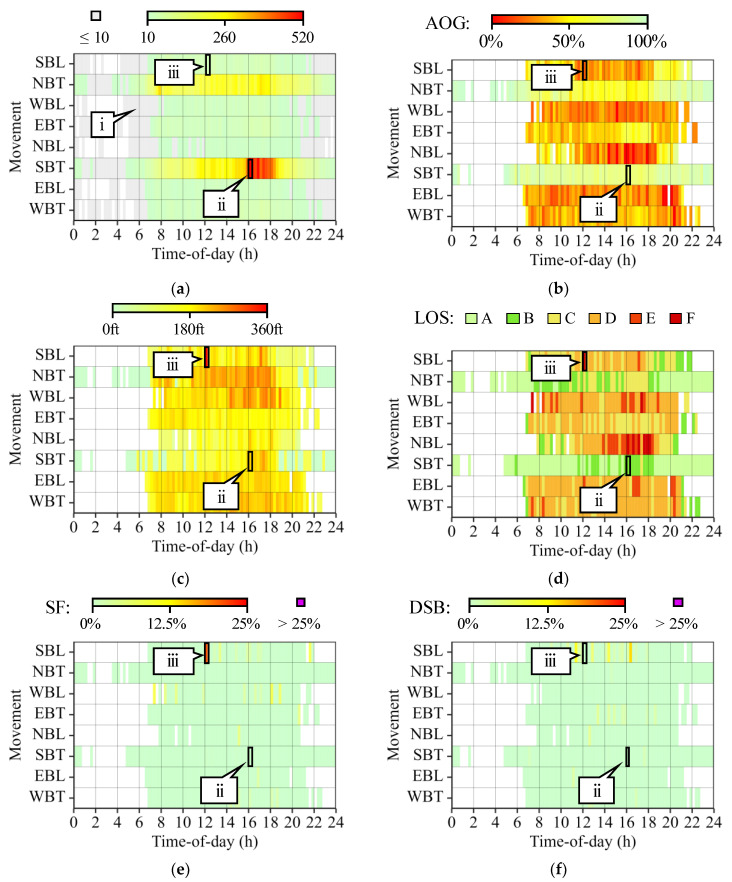
Traffic signal vehicle performance measures by movement and TOD. (**a**) Trajectory count. (**b**) AOG. (**c**) 85QL. (**d**) LOS. (**e**) SF. (**f**) DSB.

Figure 7 shows heatmaps that provide vehicle traffic signal performance results by movement and TOD period. This information is relevant to practitioners as it allows them to either confirm the correct operation of the intersection or identify the need for signal retiming or capital investment improvements [4,5,6,7,8,9,10]. It is important to note that, to improve confidence, traffic signal performance results are not provided for any 15 min period where only 10 vehicles, or fewer, were detected for an intersection movement (Figure 7a, callout i).

At this intersection, demand is highest for the SBT and NBT movements (Figure 7a), which also display the best AOG values due to corridor coordination (Figure 7b). The 85QL shows that the largest estimated queues are shorter than 360 ft. from the far side of the evaluated movements (Figure 7c). LOS is most challenging for the NBL movement from 14:00 to 18:15 h. (Figure 7d). Additionally, only moderate to minor sporadic split failure (Figure 7e) and downstream blockage (Figure 7f) events occur throughout the day.

Two movement TOD periods that have highly contrasting performance are SBT from 16:00 to 16:15 h. (callout ii) and SBL from 12:00 to 12:15 h. (callout iii). Callout ii has one of the highest trajectory counts; however, it shows high AOG and low LOS, SF, and DSB values. On the other hand, callout iii experiences challenging performance even as it only deals with moderate demand, likely due to the passing impact of lunch traffic.

**Figure 8:**
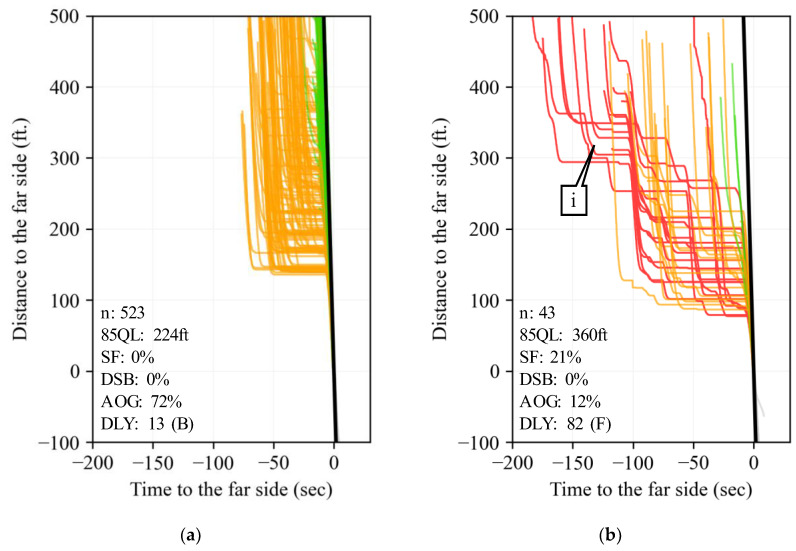
PPDs of highlighted movements and TODs in Figure 7. (**a**) Figure 7, callout ii. (**b**) Figure 7, callout iii.

Figure 8 shows the corresponding PPDs of callouts ii and iii in Figure 7. These graphics help corroborate the traffic signal conditions identified with the heatmaps. For example, it is clear that several vehicles experience split failure events at the SBL movement (Figure 8b, callout i) from 12:00 to 12:15 h.

### 3.2. Pedestrian Performance by TOD

**Figure 9:**
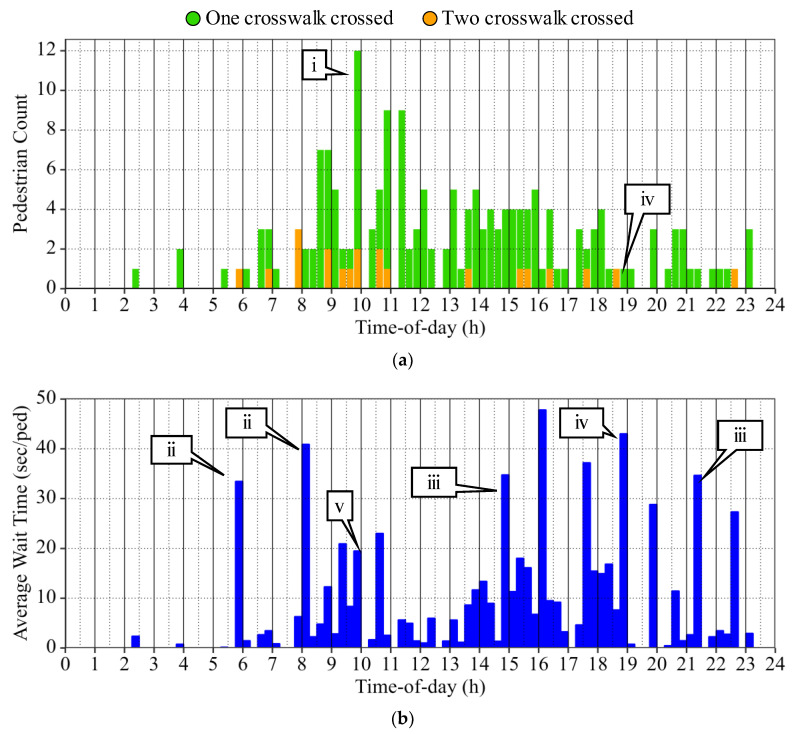
Traffic signal pedestrian performance measures by TOD. (**a**) Number of crosswalks crossed. (**b**) Average wait time.

Figure 9 shows bar graphs in 15 min intervals that display the number of pedestrians that crossed one or two crosswalks (Figure 9a) and the estimated average wait times (Figure 9b). These visualizations can be used by practitioners to verify if the pedestrian experience at the evaluated intersection is as designed, or if operational changes are required.

A peak in pedestrian volume is observed from 9:45 to 10:00 h. (Figure 9a, callout i), and the longest wait times occur from 5:45 to 8:15 h. (Figure 9b, callout ii) and from 14:45 to 21:30 h. (Figure 9b, callout iii). It is important to analyze these graphics in conjunction since average wait times have more relevance when estimated from numerous pedestrian trajectories.

For example, the second largest average wait time occurs from 18:45 to 19:00 h. (Figure 9b, callout iv); however, there was only one detected pedestrian that crossed one crosswalk during that time (Figure 9a, callout iv). Therefore, actionable conclusions may be hard to derive from this estimated wait time as it is based only on one sample. In contrast, during the peak pedestrian volume period with 12 sampled individuals (Figure 9a, callout i), there was an estimated average wait time of just under 20 s/ped (Figure 9b, callout v). This measurement is a more representative result of pedestrian experience at the analyzed intersection than those derived from fewer samples, and actions based on this wait time estimation would likely have a more meaningful impact.

### 3.3. Pedestrian Speeds

**Figure 10:**
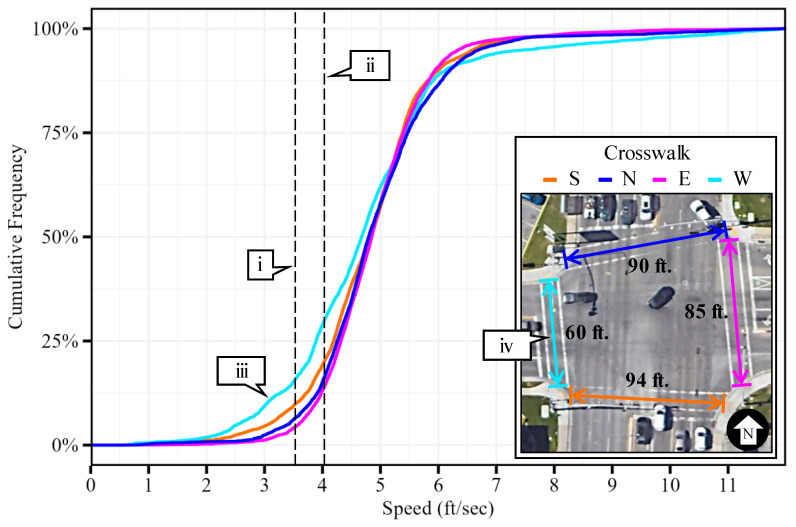
Cumulative frequency diagram of pedestrian speeds within crosswalks (map data: Google).

In addition to the estimation of traffic signal performance measures from LiDAR-derived trajectory data, the dataset can be used to corroborate state-of-the-practice design parameters. For example, the MUTCD indicates that pedestrian clearance intervals should be sufficient to allow a person traveling at 3.5 ft/s, or at 4.0 ft/s where pedestrian clearance time extension is possible, to reach the far side of the traveled way or a safe median to wait [40]. At this location, UDOT runs a pedestrian clearance time based on a 4 ft/s pedestrian speed that can be extended up to five seconds for slower traveling pedestrians [43]. Figure 10 shows cumulative frequency diagrams (CFDs) of sampled pedestrian speeds within the intersection’s crosswalks derived from over 29,000 waypoints that can be used for comparison.

The 3.5 ft/s and the 4.0 ft/s MUTCD speed guidelines [40] are marked for reference (callouts i and ii, respectively). It is shown how most pedestrian waypoints have speeds faster than these values. With an overall sampled 15th percentile speed of 3.9 ft/s, we can corroborate that the MUTCD provides conservative pedestrian speed guidelines for clearance design at the evaluated intersection.

Additionally, it can be observed that a higher proportion of pedestrians travel at slower speeds (callout iii) when traversing the west (W) crosswalk (callout iv). This is likely due to the crosswalk having the shortest length, where pedestrians likely do not feel rushed to cross the street. However, this is speculation, and further research should focus on a detailed analysis of pedestrian behavior where LiDAR sensor data are available.

## 4. Conclusions

This study presented techniques to create vehicle and pedestrian PPDs from LiDAR-derived trajectory data to estimate traffic signal performance measures. Vehicle PPDs created from over 15,000,000 waypoints were used to estimate movement level turning counts, 85th percentile queue lengths, HCM LOS, AOG, SF, and DSB of 15 min periods (Figure 7). Pedestrian PPDs created from over 170,000 waypoints were used to estimate intersection-level wait times and the proportion of people that traversed one or two crosswalks, also by 15 min periods (Figure 9). The presented approach to estimate traffic signal performance from LiDAR data provides the following benefits:

- It can be used to evaluate signal performance in real-time since LiDAR systems can sample objects with a tenth-of-a-second frequency.
- Insightful trends can easily be evaluated by expanding the analysis period. For example, weekly analyses can provide knowledge on the impact of recently implemented timing changes. Monthly analyses can provide information on the impact of long-term construction projects and seasonal changes. Yearly analyses can help identify capital investment opportunities.
- It allows for an analysis that can detect not only trends but also rare events over short periods of time since virtually all traversing objects are detected. Therefore, no data aggregation is needed to obtain accurate results.
- By fusing the detection of various LiDAR sensors, the entire progression of vehicles as they cross through the intersection can be evaluated.
- Different modes of transportation can be analyzed.

The discussed techniques can be implemented at any location that counts with LiDAR detection to provide practitioners with insights into the operational conditions of their managed intersections.

In addition to the calculation of traffic signal performance measures, LiDAR-derived data can be used to evaluate pedestrian walking speeds. For example, an analysis of the distribution of pedestrian speeds within the crosswalks (Figure 10) corroborated that the MUTCD speeds guidelines for clearance intervals are indeed conservative values at the analyzed intersection that likely provide safe timing practices. However, LiDAR data provides an opportunity to customize signal timing that better serves local demographics instead of following national trends.

Even though there are many benefits from using LiDAR data for intersection analysis, an important difficulty to consider is streamlining the extensive data outputs to facilitate agencies easily deriving outcome-oriented performance measures such as those shown in Figure 7, Figure 8 and Figure 9. As these challenges are addressed by the vendor community, we anticipate that the ability to derive real-time PPDs (such as Figure 4) for all movements will become very attractive for agencies. Furthermore, deriving summary graphics such as those shown in Figure 4 and Figure 8 provide the ability to directly compare performance measures obtained from CVs [37].

## Figures and Tables

**Table 1:** HCM LOS criteria for signalized intersections.

LOS	Average Control Delay (s/veh)	Description
A	≤10	Free flow
B	>10–20	Stable flow (slight delay)
C	>20–35	Stable flow (acceptable delays)
D	>35–55	Approaching unstable flow (tolerable delay)
E	>55–80	Unstable flow (intolerable delay)
F	>80	Forced flow (congested and queues fail to clear)

## Data Availability

The original contributions presented in the study are included in the article, further inquiries can be directed to the corresponding author.
